# Repeated Endovascular Treatment of Early Recurrent Proximal Middle Cerebral Artery Occlusion: Case Report and Brief Review of the Literature

**DOI:** 10.3389/fneur.2018.00289

**Published:** 2018-05-03

**Authors:** Simon Fandler, Hannes Deutschmann, Franz Fazekas, Thomas Gattringer

**Affiliations:** ^1^Department of Neurology, Medical University of Graz, Graz, Austria; ^2^Division of Neuroradiology, Vascular and Interventional Radiology, Department of Radiology, Medical University of Graz, Graz, Austria

**Keywords:** thrombectomy, endovascular treatment, stroke, ischemic stroke, recurrent stroke, large vessel occlusion

## Abstract

Mechanical thrombectomy (MT) is the gold standard treatment for large vessel occlusion (LVO) stroke of the anterior circulation. Whether MT can also be effectively and safely performed in early recurrent LVO is largely unclear. We present the case of a middle-aged patient who was successfully treated by MT for right proximal middle cerebral artery (MCA) occlusion with excellent outcome. One day after discharge (9 days after the first MT), the patient was readmitted with wake-up stroke. MRI again revealed right proximal MCA occlusion with severe diffusion–perfusion mismatch. Repeat MT was performed and once more led to almost full recovery. The recurrent strokes were attributed to ulcerated non-stenosing plaques in the ipsilateral internal carotid artery, which prompted thromboendarterectomy. In an 18-months follow-up period, no further vascular events occurred. In conclusion, repeated MT for early recurrent LVO appears feasible in carefully selected patients. The collection of similar cases *via* registries would be desirable.

## Background

Mechanical thrombectomy (MT) in combination with intravenous thrombolysis (IVT) has become the gold standard treatment for acute ischemic stroke due to proximal large vessel occlusion (LVO) of the anterior circulation (1). In special clinical situations where IVT is contraindicated, MT alone has also been found effective (2).

Intravenous thrombolysis is generally contraindicated in patients with a history of stroke within the last 3 months because of the assumed higher risk of intracranial hemorrhage. Especially the repeated use of IVT in early recurrent stroke might pose a risk, although a small case series has shown that repeated IVT can be safely and effectively administered in carefully selected patients (3).

In such a situation, MT might be an option. However, this has not yet been systematically evaluated and there are only few publications on repeated thrombectomies for LVO stroke (4–6). Data on repeated MT for early recurrent stroke caused by the occlusion of the same affected vessel are especially scarce.

Here, we present a patient with recurrent stroke due to occlusion of the same vessel who was successfully treated by MT twice within 9 days.

## Case Presentation

A 66-year-old retired female patient was admitted with left-sided hemiplegia, dysarthria, and hemineglect 30 min after symptom onset. The National Institutes of Health Stroke Scale (NIHSS) score was 13. Medical history included coronary heart disease, hypertension, diabetes, and smoking. Notably, she had a history of hemithyroidectomy 1 week prior because of goiter.

Acute non-contrast computed tomography (CT) was unremarkable. CT angiography revealed right-sided middle cerebral artery (MCA) M1 occlusion. IVT was contraindicated because of the recent surgery, and MT was initiated.

**Figure 1:**
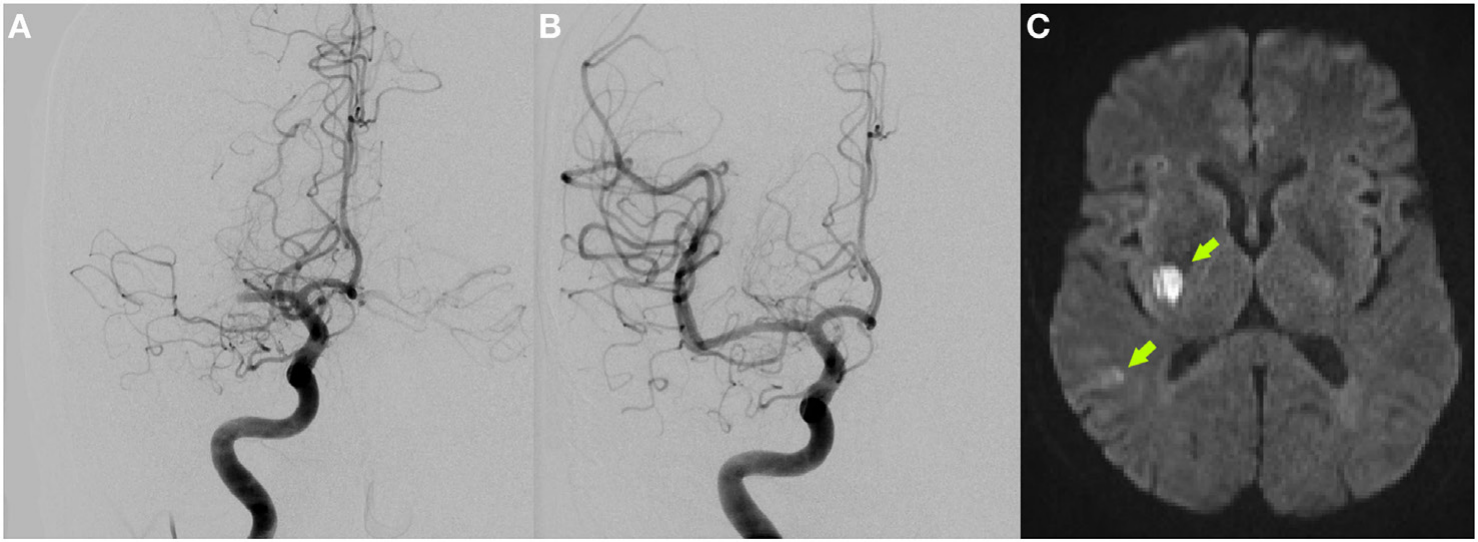
Initial digital subtraction angiography showing right proximal middle cerebral artery occlusion **(A)** and complete vessel recanalization after successful mechanical thrombectomy **(B)**. DWI-MRI 4 days after stroke depicts small ischemic infarcts in the right posterior basal ganglia and temporal cortex [arrows **(C)**].

Mechanical thrombectomy was successfully performed using a Solitaire stent retriever (thrombolysis in cerebral infarction scale 3, Figures 1A–B). Symptom-to-recanalization time was 130 min.

After the procedure, the patient showed rapid major neurological improvement. Brain MRI on day 4 demonstrated small ischemic infarcts in the right posterior basal ganglia region and right temporal cortex (Figure 1C). While thorough cardiac work-up including stroke unit ECG monitoring, 24-h ECG and transesophageal echocardiography showed no cardioembolic source, duplex sonography revealed an irregular-shaped ulcerated plaque formation in the right internal carotid artery (ICA) origin but without relevant stenosis (peak systolic velocity of 66 cm/s on doppler sonography compared to 57 cm/s contralaterally; luminal stenosis of 40% on CT angiography). Therefore, only antiplatelet and statin therapy was initiated and the patient was discharged home with an excellent outcome on day 8 [NIHSS: 0, modified Rankin scale (mRS): 1].

On the next morning (day 9 after the index stroke), she was re-admitted with wake-up-stroke (last seen well 10 h before) and had again a right total anterior circulation stroke syndrome (NIHSS: 16).

**Figure 2:**
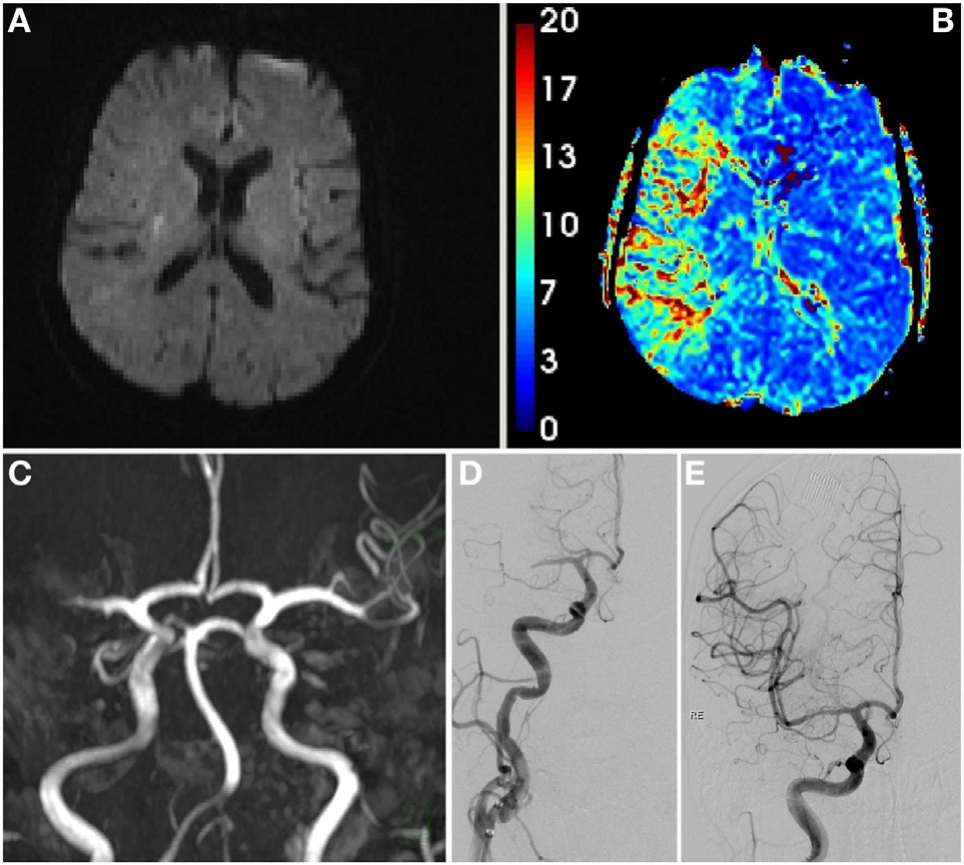
Initial MRI scan at second admission showing only a small DWI lesion corresponding to the preceding stroke **(A)**, hypoperfusion of large parts of the middle cerebral artery (MCA) territory [**(B)**, mean transit time], and right proximal MCA occlusion [**(C)**, time-of-flight angiography]. Digital subtraction angiography pre- **(D)** and post-thrombectomy **(E)**.

Multimodal MRI was performed. Aside from the past infarction, no new diffusion or FLAIR-positive lesions were found. However, a right-sided proximal MCA occlusion was present again and was associated with severe hypoperfusion (Figures 2A–C).

As thrombolysis was contraindicated (both because of recent surgery and recent stroke) and MRI showed an extensive diffusion–perfusion mismatch, MT was again successfully performed with a Solitaire stent retriever (door-to-recanalization time: 149 min, thrombolysis in cerebral infarction scale 3, Figures 2D–E).

The neurological exam on the following day showed once more remarkable recovery (NIHSS: 0). Postinterventional duplex sonography and contrast-enhanced MR-angiography did not show any signs of vasospasm or vessel dissection.

**Figure 3:**
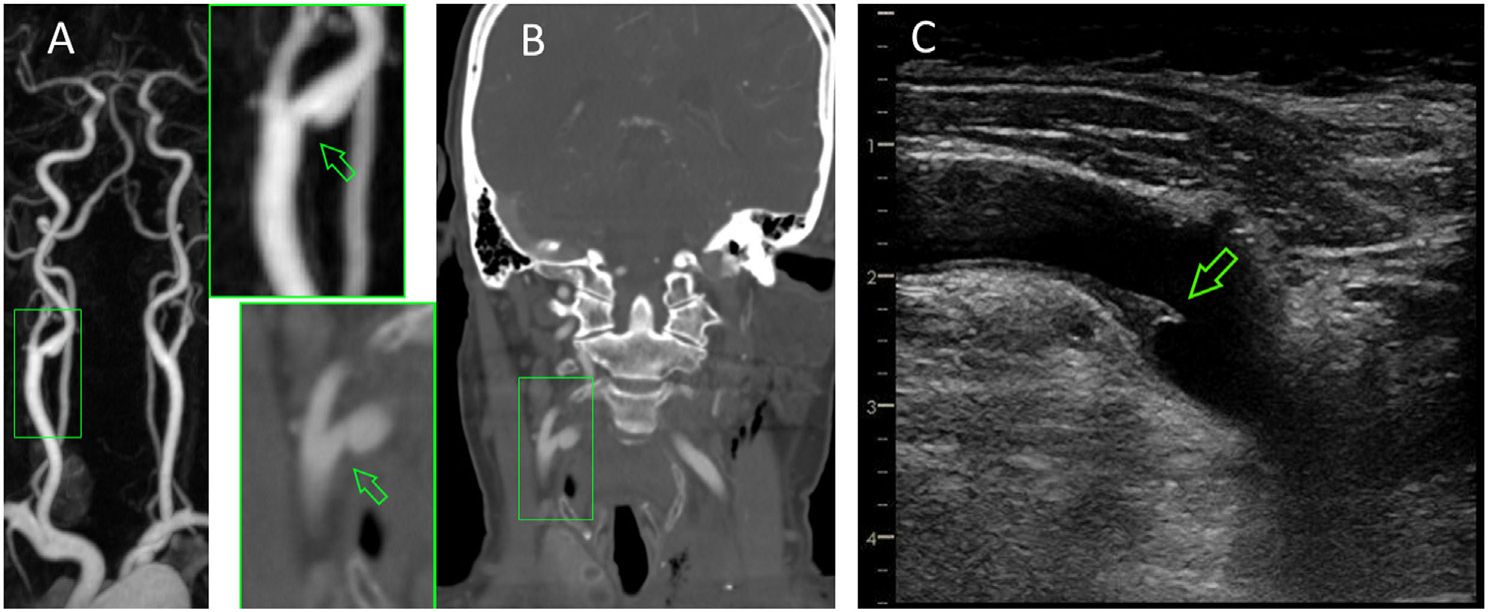
Irregular-shaped plaque formation at the origin of the ipsilateral internal carotid artery (arrows) as seen in contrast-enhanced MRA **(A)**, computed tomography angiography **(B)**, and neurosonography **(C)**.

Detailed etiological re-evaluation including CT angiography of the aortic arch, repeated echocardiography, and 24-h ECG revealed no new findings. The already known ulcerated plaque at the right ICA origin (Figure 3) was, therefore, considered as the most likely cause of the two strokes, and uneventful carotid thrombendarteriectomy was performed. The patient was discharged home with only minimal clumsiness of the left hand (NIHSS: 0, mRS: 1).

Repeated neurological follow-up examinations were performed at 3, 6, and 18 months, each showing no further vascular events and stable neurosonographic findings.

## Discussion

We here present the case of a patient with probable macroangiopathy-related recurrent severe stroke who underwent successful MT of the same occluded proximal MCA twice within a few days.

To our knowledge, there are only three publications on repeated MT for stroke within a short time frame. Those are two single case reports and one case series, the latter is composed of patients with a wide range of interthrombectomy intervals, ranging from less than 1 to 278 days (4–6). In total, eight cases treated by repeated MT in the MCA or intracranial ICA within <14 days have been reported to date. In half of these cases, re-thrombectomy was performed in the same vessel, in the other half, LVO had reoccurred contralaterally. While MT was technically successful in all these cases, patients’ outcome varied substantially (mRS at 90 days or discharge, if former not available: 0–2: *n* = 5; mRS 3–5: *n* = 0; mRS 6/death: *n* = 3).

Contrary to our patient, cardioembolism was the predominant stroke etiology in prior reports. This is the first detailed description of a macroangiopathy-related early recurrent stroke treated by re-thrombectomy.

Because repeated and extensive stroke work-up showed no indication for other etiologies, the severe ulcerated plaque formation in the ipsilateral ICA was considered the most likely cause for the recurrent strokes (7). As the patient suffered from two LVO strokes within a short time period, we decided to perform carotid thromboendoarterectomy despite missing evidence for this approach. The absence of further cerebrovascular events during follow-up is in favor of this decision. Initiation of short-term (e.g., 3 months) dual antiplatelet therapy both after the first or second stroke event would have also been an alternative treatment approach (8).

The excellent outcome after two LVO strokes can be attributed to two main reasons. At the first stroke, symptom-to-recanalization time was rather short. For the second stroke, symptom onset was unclear due to the wake-up stroke scenario. However, MRI showed no new DWI lesion despite a large perfusion deficit. This fact also argues for a short interval between stroke onset and intervention alongside the presence of a good collateral circulation. Interestingly, final infarct size after both thrombectomies was minimal.

Repeated thrombectomy may lead to more severe disruption of the vascular endothelium, thereby increasing the risk of complications such as vasospasm, arterial dissection, as well as intracranial hemorrhage (9). In our case (with an interthrombectomy interval of 9 days), no such complications occurred. In line with this finding, a previous study using 3-T vessel wall MRI conducted within 1 week after MT reported no relevant residual vessel wall injuries (10). Furthermore, it has to be noted that our patient underwent MT immediately in both situations and did not receive IVT. IVT potentially promotes blood–brain barrier disruption and neurotoxicity, and thus might increase ICH risk (11). It is, therefore, unfortunate that the previously mentioned case series did not report on concomitant IVT treatment (4).

## Concluding Remarks

Repeated MT for early recurrent LVO stroke appears feasible in carefully selected patients. However, more experience in the management of such patients is important and the collection of similar cases *via* registries would be desirable.

## Ethics Statement

Written informed patient consent for publication of this case report has been obtained.

## Author Contributions

SF and TG contributed conception and design of the study; SF wrote the first draft of the manuscript; HD, FF, and TG wrote sections of the manuscript. All authors contributed to data analysis and interpretation as well as to the manuscript revision, read, and approved the submitted version.

## Conflict of Interest Statement

The authors declare that the research was conducted in the absence of any commercial or financial relationships that could be construed as a potential conflict of interest.
